# Out of the Box Binding Determines Specificity of SH2 Domain Interaction

**DOI:** 10.1016/j.str.2009.07.002

**Published:** 2009-08-12

**Authors:** Susanne Müller, Stefan Knapp

**Affiliations:** 1 Structural Genomics Consortium, University of Oxford, Old Road Campus Research Building, Roosevelt Drive, Headington, Oxford OX3 7DQ, UK; 2 Department of Clinical Pharmacology, University of Oxford, Old Road Campus Research Building, Roosevelt Drive, Headington, Oxford OX3 7DQ, UK

## Abstract

SH2 domains are phosphotyrosine specific interaction modules with largely overlapping sequence specificities. A recent structure by Bae et al. revealed that SH2 domain specificity can be mediated by secondary binding sites located outside the phosphotyrosine binding pocket.

## Main Text

Selective recognition of phosphotyrosine (pY) signals by small modular binding domains is a fundamental principle of cellular signaling. The Src homology 2 (SH2) domain is the most prevalent of the pY specific signaling modules. The human genome contains 120 SH2 domains present in 110 signaling molecules, which play a central role in the regulation of tyrosine kinase signaling pathways (Liu et al., 2006). Directed phosphopeptide library screening revealed the sequence specificity of most SH2 domains and showed that amino acids located from −2 to +4 relative to the pY contribute to selectivity and high affinity binding of peptidic substrates (Songyang et al., 1993). However, these studies also revealed that a multitude of pY-containing sequences can be recognized by several SH2 domains with similar affinity in vitro. Many signaling molecules contain more than one interaction module, suggesting that cooperative binding of several binding events will provide selectivity of signaling in vivo. However, trying to explain the selectivity of signaling processes with the specificity of SH2 domain pY binding sites would be an oversimplification of the complex interactions in cellular signalosomes.

The family of fibroblast growth factor receptors (FGFRs) represents four receptor tyrosine kinases that recruit a number of signaling partners to the plasma membrane as a response to FGF stimulation. A key FGFR interaction partner is phospholipase Cγ (PLCγ), which interacts via its SH2 domain with the highly conserved C-terminal tail residue Y766 (FGFR1). However, PLCγ possesses two SH2 domains (N-SH2 and C-SH2: N- and C-terminal SH2 domains) that recognize the same substrates and have similar binding affinity for pY peptides derived from the FGF1R tail as well as other sites located in PDGFR and other RTKs (Ji et al., 1999). Binding studies in growth factor stimulated cells showed that only the PLCγ N-SH2, but not C-SH2, binds to pY sites present in RTK tails, whereas the C-SH2 binds intramolecularly to pY783 stimulating phospholipase activity by stabilizing a structural rearrangement in PLCγ (Poulin et al., 2005).

Interestingly, the recent crystal structure of a complex of the two PLCγ SH2 domains with active FGFR1 showed that a high affinity interaction of N-SH2 with FGFR1 is mediated by a secondary binding site located outside the SH2 domain pY binding site (Bae et al., 2009). Bae et al. (2009) showed, by a combination of structural, mutagenesis, and binding studies, how the SH2 domain selectivity is regulated in vivo to mediate a specific cellular process and explains the selectivity for N-SH2 for the FGFR1 C-terminal tail.

The detailed structural analysis revealed that, as expected, the N-SH2 domain is bound to the pY^766^LDL sequence within the C-terminal tail of FGFR1-3P, whereas the C-SH2 binding site is unoccupied and exposed to the solvent, allowing additional intramolecular interaction with pY783 upon the PLCγ activation. The identified secondary binding site is located distantly from the N-SH2 pY site interacting with the C-terminal kinase lobe. In N-SH2, the secondary interaction site comprises residues located in the stands βD, the loops BC and DE interacting with FGF1R residues in the helices αE and αI, and the sheet β8. This interaction covers 533 Å^2^, a surface area that is comparable with the area covered by the primary pY interaction site. The secondary pocket consists of two subsites with different surface properties. The first subsite is mainly hydrophobic and forms several van der Waals contacts with FGFR1 residues V758, A759, V636. and hydrophobic areas of the sidechains Q606 and D755. The second subsite contains mainly charged residues forming polar interactions with FGF1R, as exemplified by polar interactions between R609 in FGFR1 αE with PLCγ D594 and the carbonyl oxygen of S612. Importantly, in contrast to the pY binding sites, residues located in the secondary binding site of N-SH2 that mediate interaction with FGF1R are highly divergent from residues in corresponding positions in C-SH2, explaining the observed selectivity for the N-terminal domain in vivo.

The presence of the secondary binding site in N-SH2 also increases the binding strength significantly. Isothermal titration calorimetry experiments showed that the N-SH2 binds 10–40 times more tightly to the kinase domain of activated FGF1R (FGF1R-3P) than to pY peptides lowering the dissociation constant to 33 nM. Importantly, mutagenesis experiments revealed that interactions mediated by the secondary binding site are required for stimulation of phosphatidylinositol-4,5-biphosphate hydrolysis in FGF-stimulated cells.

Bae et al. (2009) report an important finding that provides additional insight and enhances our understanding of the specificity of pY signaling cascades and demonstrates that SH2 domains are versatile and complex signaling modules that can also mediate interactions outside their pY binding pocket. Furthermore, recent structural studies revealed that interactions mediated outside the pY pocket of SH2 domains are essential for maintaining the active state of the tyrosine kinases Csk, cAbl, and Fes (Filippakopoulos et al., 2008; Ogawa et al., 2002). These observations open an interesting area for future structural and functional studies that will elucidate the molecular mechanisms that determine the high degree of specificity of in vivo signaling events.

## References

[bib1] Bae J.H., Lew E.D., Yuzawa S., Tome F., Lax I., Schlessinger J. (2009). Cell.

[bib2] Filippakopoulos P., Kofler M., Hantschel O., Gish G.D., Grebien F., Salah E., Neudecker P., Kay L.E., Turk B.E., Superti-Furga G. (2008). Cell.

[bib3] Ji Q.S., Chattopadhyay A., Vecchi M., Carpenter G. (1999). Mol. Cell. Biol..

[bib4] Liu B.A., Jablonowski K., Raina M., Arce M., Pawson T., Nash P.D. (2006). Mol. Cell.

[bib5] Ogawa A., Takayama Y., Sakai H., Chong K.T., Takeuchi S., Nakagawa A., Nada S., Okada M., Tsukihara T. (2002). J. Biol. Chem..

[bib6] Poulin B., Sekiya F., Rhee S.G. (2005). Proc. Natl. Acad. Sci. USA.

[bib7] Songyang Z., Shoelson S.E., Chaudhuri M., Gish G., Pawson T., Haser W.G., King F., Roberts T., Ratnofsky S., Lechleider R.J. (1993). Cell.

